# Spanish Version of the Caregiver Contribution to Self-Care of Heart Failure Index (CC-SCHFI): A Psychometric Evaluation

**DOI:** 10.3390/jpm12040625

**Published:** 2022-04-12

**Authors:** Rosa Antonio-Oriola, Ercole Vellone, Angela Durante, Maddalena De Maria, Marco Di Nitto, Vicente Gea-Caballero, Iván Santolalla-Arnedo, Michał Czapla, José Vicente Benavent-Cervera, Juan Luis Sánchez-González, Raúl Juárez-Vela

**Affiliations:** 1 Doctorate Program in Clinical and Community Nursing, University of Valencia, 46001 Valencia, Spain; antonio.ros81@gmail.com; 2 Department of Biomedicine and Prevention, University of Roma Tor Vergata, 00133 Rome, Italy; ercole.vellone@uniroma2.it (E.V.); maddalena.demaria@outlook.it (M.D.M.); 3 Group of Research in Care GRUPAC, Department of Nursing, University of La Rioja, 26004 Logroño, Spain; ivan.santolalla@unirioja.es (I.S.-A.); michal.czapla@umw.edu.pl (M.C.); raul.juarez@unirioja.es (R.J.-V.); 4 Centro per l’Eccellenza Clinica, la Qualità e la Sicurezza Delle Cure (CNEC), Istituto Superiore di Sanità, 00162 Rome, Italy; marco.dinitto@iss.it; 5 Faculty of Health Sciences, Valencia International University, 46002 Valencia, Spain; vagea@universidadviu.com (V.G.-C.); josevicente.benavent@campusviu.es (J.V.B.-C.); 6 Laboratory for Experimental Medicine and Innovative Technologies, Department of Emergency Medical Service, Wroclaw Medical University, 51-616 Wroclaw, Poland; 7 Faculty of Nursing and Phisiotherapy, University of Salamanca, 37007 Salamanca, Spain; juanluissanchez@usal.es

**Keywords:** caregivers, self-care, heart failure, psychometrics, validity, reliability

## Abstract

Background: The Caregiver Contribution to Self-Care of Heart Failure (CC-SCHFI) is a theoretically driven instrument to measure the extent to which caregivers support heart failure (HF) patients to perform self-care. The CC-SCHFI measures caregivers’ contribution to self-care maintenance and self-care management and caregiver confidence in contributing to heart failure patients’ self-care. To date, the CC-SCHFI has never been tested in Spanish-speaking populations. Purpose: To translate the CC-SCHFI from English into Spanish and to test its psychometric characteristics. Method: CC-SCHFI translation and back-translation were performed according to the Beaton et al. methodology. Data from a cross-sectional study conducted in an outpatient clinic in Spain were used for the analysis. Psychometric analysis was performed with exploratory factor analysis (EFA) with oblique rotation. Results: Caregivers had a mean age of 60.5 years (SD 14,9) and the majority were female (85%). Data from 220 caregivers were analyzed. From EFA, using the principal axis factoring method, we extracted two factors in the self-care maintenance subscale (“treatment adherence behaviors” and “symptom control and maintenance behaviors”), two in the self-care monitoring subscale (“illness behaviors” and “prevention behaviors”) and one factor for the self-efficacy subscale. The Pearson’s rank correlation coefficients between SCHFI and CCSCHFI showed significant correlation in each subdimension.

## 1. Introduction

Heart failure (HF) is a common and serious health problem and has been defined as a global pandemic, affecting around 26 million people worldwide [1,2]. An estimated 1 to 2% of the European population suffers from HF, with an increasing prevalence largely due to population aging [2] and an annual mortality rate in patients admitted to hospital of between 15 and 45% [3]. Updated data on medium- and long-term prognosis based on the registry of the European Society of Cardiology ESC-EORP-HFA Heart Failure Long-Term highlight its morbidity and mortality [4].

HF is a significant health problem in Spain, as in many other European countries, with prevalence ranging between 4.7–6.8% [5]. HF prevalence increases exponentially with age and doubles with each decade to over 8% among those over 75 years [6]. This increase leads to an important degree of comorbidity and frailty [7,8,9], which affect quality of life compared to the general Spanish population or other chronic diseases [10]. HF is also the leading cause of hospitalization in people over 65 years of age in Spain, accounting for 7.1% of public health expenditure [11], most of which is related to hospital admissions [12]. The rate of readmission due to heart disease in Spain is 32.6% [5]. Furthermore, a general increase in the rate of frequency was observed between 2003 and 2018 in the population aged 80 years or older [13].

Interventions to promote self-care have been shown to have a positive impact on several outcomes, such as: length of stay; all-cause death; and quality of life [14]. Self-care in HF patients has been defined as a naturalistic decision-making process that is used to maintain physiological stability (self-care maintenance) and to control symptoms when they occur (self-care management) [15,16]. Guidelines from the most important cardiac societies [17,18] recommend interventions that improve self-care by increasing knowledge of HF, supporting patients, and including caregivers in acquiring self-care skills. In addition, HF treatment and self-care become more difficult with aging, since frailty is common in older HF patients; a recent study indicates that it may be present in more than 70% of patients with HF older than 80 years [19]. Thus, older adults with HF can have lower levels of self-care and need caregiver interventions to promote and improve self-care behaviors. Indeed, interventions to promote self-care should include not only patients but also caregivers, as they are the main support perceived by patients [20].

The caregiver’s contribution to self-care is crucial in the management of patients with HF, as their presence is associated with a positive prognosis and reduced use of hospital services [17,21,22,23]. In contributing to HF self-care, caregivers adapt their behaviors to the patient’s ability to perform self-care. In some cases, they only make recommendations on control and maintenance measures [24]. However, when patients are unable to practice self-care for whatever reason, caregivers are a substitute for patients in all self-care processes [25]. Care provided by non-professionals in the family and affective environment plays a key role in the social support network. In 2008, it was estimated that the hours of informal care provided in Spain to dependent people aged 65 and over amounted to 3249 million, with monetary values ranging from EUR 24,918 to 41,291 million [26].

One instrument used to measure caregiver contribution to HF patient self-care is the Caregiver Contribution to Self-Care of Heart Failure Index (CC-SCHFI), an instrument derived from the Self-Care of Heart Failure Index Version 6.2. The CC-SCHFI, developed by Italian researchers, measures caregivers’ contribution to the self-care maintenance and management of HF patients, as well as their confidence in their ability to contribute to the self-care of HF patients [25]. The availability for clinical practice of an instrument such as the CC-SCHFI, culturally adapted to other countries and cultural settings, can help in the definition and adaptation of care plans, allowing a reliable assessment of the caregiver’s contribution to the patient’s self-care process.

For this reason, the purpose of our study was to translate and adapt the English version of the CC-SCHFI to Spanish, in order to evaluate the psychometric properties of the CC-SCHFI in a sample of caregivers of people with HF.

## 2. Materials and Methods

This is a secondary analysis of a cross-sectional study. Data were collected on a convenience sample of 220 caregivers of people with heart failure, from February to December 2017 in Spain.

### 2.1. Translation, Adaptation, and Modeling

A process of cross-cultural adaptation of the CC-SCHFI was carried out from its original English version to Spanish according to Beaton et al. [27], which divides the process into six stages: (1) initial translation, (2) synthesis of translations, (3) back translation, (4) expert committee evaluation, (5) testing the draft version, (6) submission of the final version to the developers or Coordinating Committee for the Evaluation of the Adaptation Process. The Spanish translation endorsed by the original author is available at https://self-care-measures.com/ (accessed on 3 February 2022).

As established by this methodology, the original CC-SCHFI was translated into Spanish by a bilingual researcher familiar with the concepts examined by the questionnaire. The next step was the reverse translation, which was done by a bilingual researcher who was totally blind to the original version and without knowledge of the concepts explored, who translated the questionnaire into the original language (English).

Both researchers were aware of the method and in particular to use simple sentences and to avoid metaphors, colloquial terminology, passive sentences, and hypothetical statements to produce a more reliable equivalence. To verify that the items still maintained conceptual equivalence, the back-translated version of the CC-SCHFI was shared with the authors of the original instrument, and a final Spanish version of the CC-SCHFI was established after minor adjustments were resolved by email. To achieve cross-cultural equivalence, a committee of experts compared and contrasted the original and back-translated versions of the CC-SCHFI and agreed, by consensus, on a final Spanish version of the CC-SCHFI. The aim of the expert committee was to adapt the Spanish version of the CC-SCHFI as accurately as possible to obtain equivalence between the source and target versions. The committee was composed of native teachers in both languages with clinical experience involved in the process.

Finally, cognitive interviews were completed to assess comprehension and applicability in a convenience sample of 32 informal caregivers. In this phase, minor changes were made to the translation to improve the readability of the items. Some examples of physical activity (i.e., gardening and cleaning) were added to clarify differences between items measuring “exercise” and “physical activity”.

### 2.2. Data, Setting and Sample

The study was carried out in Aragon (Spain) using a cross-sectional design through non-probabilistic convenience sampling. For the calculation, the recommendations for this type of study were considered, which indicated recruiting between 5 to 10 participants per item, with a minimum of 200 [28]. The sample was *n* = 220 informal caregivers of patients admitted to the Hospital Clinic Lozano Blesa in Zaragoza (Spain), who met the following inclusion criteria: (1) being the primary caregiver of a person with HF according to the criteria of the European Society of Cardiology (ESC) [17], and (2) being 18 years of age or older. We excluded caregivers of patients with deficits in cognitive or communication skills. Data collection was performed during patients’ admission by a qualified research nurse, who had been trained specifically for this project. Caregivers were interviewed once they had given their informed consent to participate in the study.

All participants completed the Spanish version of the CC-SCHFI, which comprises its three scales, i.e., the self-care maintenance scale (10 items), the self-care management scale (6 items) and the self-care confidence scale (6 items). Each item uses a four-point Likert scale for responses, with a standardized score from 0 to 100 where higher scores indicate a greater contribution to self-care. A sociodemographic questionnaire was also used to collect characteristics and factors related to HF such as age, gender, marital status and level of education, current job, number of children, whether living with the patient, and number of hours of care per day.

### 2.3. Statistical Analysis

Descriptive statistics were computed for all variables (frequency, percentage, mean, standard deviation (SD), kurtosis, and skewness coefficients where appropriate). The Kaiser–Meyer–Olkin measure of sampling adequacy and Bartlett test of sphericity were used to assess data factorability. Bartlett’s test of significant sphericity (*p* < 0.0001) and KMO index < 0.50 indicate an adequate sample to support factor analysis [29].

Since the CC-SCHFI is a theory-based instrument, to test its dimensionality a confirmatory approach was used. Specifically, confirmatory factor analysis (CFA) was performed for testing the factorial structures of CC-SCHFI identified in previous studies [30,31,32]. Three separate CFAs, one for each scale (CC self-care maintenance, CC-self-care management and CC-self-confidence), were performed to confirm the dimensionality of the CC-SCHFI. As items were non-normally distributed, we used the Maximum Likelihood Robust estimator, a method of parameter estimation with robust standard errors. In CFA, to examine the adequacy of the tested models, a multifaceted approach was adopted, and the following fit indices and criteria were evaluated: Comparative Fit Index (CFI), Tucker–Lewis Index (TLI), Root Mean Square Error of Approximation (RMSEA), and Standardized Root Mean Squared Residual (SRMR) [24,33].

CFI and TLI were used to compare the model of interest with a null model [34] and values of 0.90 or higher indicate a good fit [21]. RMSEA was used to evaluate lack of model fit, wherein values of 0.05 indicate a well-fitting model, values between 0.05 and 0.08 indicate moderate fit and values 0.10 indicate a poor fit [35]. SRMR was used to measure the fit in the sample, wherein values of 0.08 indicate a good fit. The traditional chi-square statistic was also interpreted together with the above indices.

Considering that culture can influence scale dimensionality [36,37], we planned to conduct exploratory factor analysis (EFA) in case of CFA misfit. Specifically, the principal axis factorization was used for EFA. Since the CC-SCHFI factors were theoretically correlated, the oblique rotation (promax rotation) [38], that assumes factor correlation, was implemented.

In EFA, to determine the number of factors to retain, multiple criteria were used, such as the simplicity of the solution (factor loadings ≥ 0.30 and no cross-loadings), examination of eigenvalues >1, the interpretability of the factor structure (Thurstone, 1947) [39], and the theoretical sense of the factor (Comrey and Lee, 1992) [40]. To decide the final factorial structure of the CC-SCHFI, the interpretability of the solution and the theory underlying the instrument [32] were also considered. The factor labels were defined by critically analyzing the factor loadings and the item contents according to similarity in meaning (Comrey and Lee, 1992) [40].

Internal consistency reliability was determined by calculating Cronbach’s alpha coefficient (0.838) [41]. Construct validity was estimated following Terwee’s recommendations [42,43]. We tested the follow theoretical hypotheses: (1) if CC-SCHFI measures the concepts described in the theoretical framework, CC-self-care confidence will be significantly associated with CC-self-care maintenance and CC-self-care management; (2) if CC-SCHFI measures the caregiver contribution to patient self-care, significant associations will exist between CC and self-care and caregiver preparedness, which we measured with the Caregiver Preparedness Scale [44].

Statistical analysis was performed using SPSS and Mplus Version 8.1 (Muthén and Muthén, Los Angeles, CA, USA).

## 3. Results

The majority of caregivers recruited were women with a mean age of 60.5 years. Educational levels were equally distributed in the sample with a slightly higher (38.2%) primary education subgroup. The majority (70.6%) of caregivers were married with two children (46.4%), did not work (54.1%), and lived with the patient (51.4%). The caregivers spent an average of 8.68 h per day caring for the patient (Table 1).

### 3.1. Descriptive Analysis of the CC-SCHFI Items

Mean, standard deviation (SD), skewness, and kurtosis for the Spanish version of the CC-SCHFI are reported in Table 2. Regarding the caregiver contribution to self-care maintenance scale, the items with the highest scores were “go to the doctor or nurse”, “use a system to help you remember medication” and “try to avoid illness”, while the items “exercise for 30 min” and “participate in physical activities” scored the lowest. On the scale of caregiver contribution to self-care management, the item “call the doctor or nurse” in the case of symptoms scored the highest, while the items “take an additional diuretic pill” and “reduce fluid intake” scored the lowest. Finally, regarding caregiver confidence in contributing to the self-care subscale, the highest-scoring item was number 18, assessing confidence in following treatment advice, and the lowest-scoring item was number 22, assessing how well a remedy works.

### 3.2. Psychometric Evaluation of the CC-SCHFI

#### 3.2.1. Dimensionality

##### Caregiver Contribution to Self-Care Maintenance Scale

The Bartlett test of sphericity for self-care maintenance was significant (*p* < 0.001), and the KMO index of sampling adequacy was 0.726. Based on these results, the data were suitable for factor analysis. Indices of skewness revealed that not all the items followed a perfect normal distribution (Table 2).

The CFAs were performed by replicating the three different factorial structures of the self-care maintenance scale previously tested in other studies (PMID: 34246255, PMID: 32511111, PMID: 33445459). All yielded inadequate adaptation indices (Table 3). For this reason, we performed an EFA. The number of eigenvalues > 1 computed by EFA totaled two (Table 4). The Caregiver Contribution to Self-care Maintenance Scale was bidimensional. The first factor was labeled CC to treatment adherence behaviors, was loaded by 8 items and explained 27.5% of the total variance; the second factor was labeled CC to health promoting exercise behaviors, was loaded by 4 items and explained 10.8% of the total variance. The two factors were significantly correlated (r = 0.301, *p* < 0.001). Table 4 shows factor loadings for each item.

##### Caregiver Contribution to Self-Care Management Scale

The Bartlett test of sphericity for self-care maintenance was significant (*p* < 0.001), and the KMO index of sampling adequacy was 0.599. All the factorial structures previously identified (PMID: 34246255, PMID: 32511111, PMID: 33445459) and replicated with CFA yielded inadequate fit indices. The number of eigenvalues > 1 computed by EFA suggest the existence of two factors (Table 4). The Caregiver Contribution to Self-care Management Scale was bidimensional. The first factor was labeled CC to Illness behaviors, was loaded by 3 items and explained 32.4% of the total variance; the second factor was labeled CC to prevention behaviors, was loaded by 3 items and explained 17.8% of the total variance. The two factors were significantly correlated (r = 0.247, *p* < 0.001).

##### Caregiver Contribution to Self-Care Confidence Scale

The Bartlett test of sphericity for caregiver contribution to self-care confidence was significant (*p* < 0.001), and the KMO index of sampling adequacy was 0.816. All the factorial structures were previously identified (PMID: 34246255, PMID: 32511111, PMID: 33445459) and replicated with CFA. The factorial structure of the self-care confidence scale identified in the Spanish sample of HF patient of Self Care of Heart Failure Index v.6.2 (SCHFI v.6.2) produced a good fit (Table 3). Thus, the caregiver contribution to the self-care confidence scale presented a unidimensional factorial structure.

Factor 1 of the Caregiver Contribution to Self-care Maintenance Scale: CC to treatment adherence behaviors.

Factor 2 of the Caregiver Contribution to Self-care Maintenance Scale: CC to health promoting exercise behaviors.

Factor 1 of the Caregiver Contribution to Self-care Management Scale: CC to illness behaviors.

Factor 2 of the Caregiver Contribution to Self-care Management Scale: CC to prevention behaviors.

#### 3.2.2. Construct Validity

The correlation coefficients of CC-self-care confidence versus CC-self-care maintenance and CC-self-care management were 0.431 and 0.479, respectively (Table 5).

Significant and positive associations were also found between each score of SCHFI and CC-SCHFI (respectively, self-care maintenance r = 0.341, *p* < 0.001; self-care management r = 0.289, *p* < 0.05; self-care confidence, r = 0.314 *p* < 0.05), Table 4. Finally, significant and positive associations were found between each CC-SCHFI score and the caregiver preparedness score (respectively, self-care maintenance r = 0.436, *p* < 0.001; self-care management r = 0.304, *p* < 0.05; self-care confidence r = 0.394, *p* < 0.001).

## 4. Discussion

The aim of this study was to carry out the cross-cultural adaptation and validation of the Caregiver Contribution to Self-Care in Heart Failure Index (CC-SCHFI) in Spanish. We then evaluated the psychometric properties, the results of which showed a valid instrument, thus obtaining a culturally equivalent instrument to measure the contribution of caregivers to the self-care of people with HF. To our knowledge, this is the first study to validate the CC-SCHFI in Spanish. To date, similar validation studies have been conducted in Brazil [30] and Thailand [31].

During the cross-cultural adaptation process, minor changes were made to correct readability, and examples were added to differentiate “exercise” from “physical activity”, modifying some terms and expressions to ensure cultural equivalence.

Psychometric analysis was performed with exploratory factor analysis (EFA) for each separate CC-SCHFI scale (caregiver contribution to self-care maintenance, caregiver contribution to self-care management, and caregiver confidence in contributing to self-care). From EFA, we extracted two factors on the self-care maintenance subscale, two on the self-care follow-up subscale, and one factor for the self-efficacy subscale. The Pearson rank correlation coefficients between SCHFI and CCSCHFI showed a significant correlation to medium/fair in each subdimension that determined the validity coefficient.

The analyses showed, as in the original study, that the Spanish version of the CC-SCHFI had a complex structure [25] that was similar to the Spanish version of the SCHFI 6.2 for patients [15,32], reflecting different aspects related to self-care. All three scales generally showed high factor loadings, but caregiver trust was the one with the highest factor loading.

In the caregiver’s contribution to self-care maintenance scale, two factors were identified, which we called “treatment adherence behaviors” and “symptom control and maintenance behaviors”. Items related to exercise and physical activity were separated from the other items, all of which are self-care activities that people should perform. The caregiver cannot replace the patient as in other actions, but recommend and remind him how important it is to perform them for his maintenance, which implies difficulty.

The factor analysis of the caregiver’s contribution to the self-care management scale identified two factors that we named “illness behaviors” and “prevention behaviors”. In the first factor we grouped activities that should respond to symptoms: “reducing fluid intake and/or increasing the dose of diuretics” and “assessing the effectiveness of treatment” are actions that involve difficulty for the caregiver since care management recommendations are not a common practice in Spain [43].

The second factor covers those prevention actions where caregivers do not see the difficulty in putting them into practice (e.g., “reducing salt intake”, “contacting the doctor or nurse for help” and “think about the person you care for in the last month, have they had difficulty breathing or swollen ankles?”). This may be because the health system in Spain is easily accessible when people or their carers need to get help [45].

In the confidence scale of the caregiver’s contribution to self-care index, we found a unidimensional structure as in the Thai version [31], which differs from the original study [46] and the Brazilian study [30]. However, curiously, the items with scores higher were those activities directed by the health team, while autonomous preventive behaviors that describe activities that require the competence of the caregiver to make decisions were lower.

An expected result was the scores of the individual items of the CC-SCHFI. In the scale of contribution to the maintenance of self-care, the item with the highest score was “going to the doctor or nurse”, while the item with the lowest score was “exercise for 30 min”, as in the Spanish version of the SCHFI 6.2 for patients [32].

These data show that informal caregivers do not adequately manage the disease, do not give their relatives self-care advice (“walk for 30 min”, “do not eat salt”, etc.), but when there is a problem, they directly call the doctor or nurse, showing a low level of self-care of disease management compared to other countries [47].

Our findings are in line with those reported by Juárez et al. [47] who evaluated the level of self-care in people with HF in Spain. However, this could also be due to the patient’s disregard of the caregiver’s recommendations. This could be explained if we take into account the cultural habits and the profile of the caregivers; our sample was mostly women with a mean age of 60.5 years, who lived with the patient (54.1%) The family structure in this context and in Spain is stereotypically that of the woman who carries out all the household tasks and serves the members living in the home, so their recommendations are generally not taken into consideration [48]. This leads them to substitute for the patient in all those activities which they are able to do, except for those which the patient has to do for themselves, which would explain the grouping of the items in our results.

There is currently no scale in Spain that measures the contribution of the caregiver of people with heart failure. The CC-SCHFI is an easy-to-administer tool that can help informal caregivers and the health care team to identify gaps in self-care, allowing the design of individual plans aimed at expanding knowledge in order to improve their skills. In addition, the CC-SCHFI has the advantage of being applied in combination with the heart failure self-care index, which is adapted to Spanish [32], making it possible to investigate the influence of reciprocity and its dimensions on the self-care of the patient–caregiver dyad, as has been studied in other countries, with the aim of being able to plan actions to improve the self-care of the HF patient [49,50].

## 5. Limitations and Strengths

The type of sampling used in the study could lead to the appearance of a Berkson bias, since all the caregivers were in the hospital because the person they care for was hospitalized recently or at the moment of the data collection; thus, it could be that they were the ones with the more advanced disease and lower levels of self-care. Nevertheless, and given the tendency for people with heart failure to be admitted and readmitted, we believe that the possibility of the Berkson fallacy appearing is remote.

This is the first study in Spanish that approximates the psychometric properties of the CC-SCHFI carried out through the official version of the CC-SCHFI.

## 6. Conclusions

Our study has shown that the Spanish version of the CC-SCHFI has a factorial validity and could be used in clinical practice and research to measure the contribution to self-care in patients with heart failure.

## Figures and Tables

**Table 1:** Sample socio-demographic characteristics (*n* = 220).

	*n*	(%)
Age (mean, SD)	60.5	(14.9)
Gender		
Men	33	(15)
Women	187	(85)
Marital Status		
Single	385	(17.3)
Married	168	(76.4)
Separated/divorced	11	(5)
Educational level		
Primary Education	84	(38.2)
Secondary Education	35	(15.9)
Vocational Training	40	(18.2)
Baccalaureate	40	(18.2)
I am currently working		
Employee	70	(33.2)
Self-employed	8	(3.6)
Pensioner	83	(37.7)
Stop	36	(16.4)
Children		
0	45	(20.5)
1	54	(24.5)
2	102	(46.4)
3	11	(5)
4	5	(2.3)
5	2	(0.9)
Lives with the patient		
No	102	(46.4)
Yes	113	(51.4)
Hours of care per day		
mean (SD)	12.31	(8.68)

**Table 2:** Descriptive statistics of the Caregiver Contribution to Self-Care of Heart Failure Index (CC-SCHFI v.1) Items.

Caregiver Contribution to Self-Care Maintenance Scale	Mean	(±) SD	Skewness	Kurtosis
How often do you recommend the following things to the patient you care for?				
1. Weigh the patient	2.56	1.68	0.37	−1.60
2. Check if the ankles are swollen	3.17	1.65	−0.27	−1.60
3. Attempt to avoid further illnesses (i.e., get a flu vaccine, avoid contact with people who are ill)	3.71	1.75	−0.77	−1.28
4. Engage in physical activity (i.e., gardening, household chores)	1.97	1.30	1.14	0.13
5. Attend appointments with the doctor or the nurse	4.32	1.35	−1.83	1.74
6. Maintain a low-sodium diet	3.20	1.57	−0.26	−1.46
7. Exercise for 30 min	1.64	1.23	1.88	2.21
8. Remember to take their medicines	2.94	1.82	0.02	−1.85
9. Order low-sodium foods when eating out or visiting other people	2.33	1.52	0.60	−1.20
10. Using a system (i.e., pill box, reminders) to help them remember to take their medicines	3.93	1.61	−1.09	−0.59
Caregiver contribution to self-care management scale ^†^				
11. If the person you are caring for has had breathing problems or swollen ankles, how likely is it that you would (or would not) recommend any of these measures?	2.44	2.30	0.10	−1.75
12. Reduce salt intake in the diet	2.84	1.77	0.06	−1.64
13. Reduce fluid intake	1.79	1.36	1.43	0.50
14. Take an extra diuretic tablet pill	1.72	1.40	1.61	0.88
15. Contact the doctor or nurse for help	4.45	1.11	−2.28	4.24
16. Think of a measure or remedy that you applied the last time the person you looked after experienced difficulty breathing or swollen ankles. Were you sure that the measure applied was helpful (or not helpful)?	2.15	1.79	0.13	−1.37
Caregiver confidence in contributing to self-care scale				
17. Keep them free of heart failure symptoms	3.13	1.50	−0.39	−1.07
18. Follow their treatment	3.57	1.52	−0.73	−0.96
19. Assess the importance of symptoms	3.28	1.33	−0.52	−0.83
20. Recognize changes in their health state should they occur	3.32	1.38	−0.60	−0.90
21. Do something to alleviate their symptoms	2.45	1.44	0.40	−1.32
22. Assess how a measure works	2.07	1.41	0.87	−0.76

^†^ The self-care management scale can only be completed if the caregiver reported symptoms within the past month.

**Table 3:** Goodness of fit indices for the previous CC-SCHFI factor models tested via CFA.

Models/Fit Indices	χ^2^ (*p*-Value)	DF	CFI	TLI	RMSEA (90% C.I.), *p*-Value	SRMR
Model 1						
CC-Self-care maintenance scale	198.689 (<0.001)	35	0.653	0.554	0.146 (0.127–0.167), <0.001	0.093
CC-Self-care management scale	81.521 (<0.001)	9	0.587	0.311	0.241 (0.194–0.290), <0.001	0.112
CC-Self-care confidence scale	125.144 (<0.001)	9	0.786	0.644	0.243 (0.206–0.282), <0.001	0.093
Model 2						
CC-Self-care maintenance scale	142.112 (<0.001)	30	0.763	0.644	0.131 (0.110–0.153), <0.001	0.073
CC-Self-care management scale	81.521 (<0.001)	9	0.587	0.311	0.241 (0.194–0.290), <0.001	0.112
CC-Self-care confidence scale	62.153 (<0.001)	9	0.902	0.837	0.165 (0.127–0.204), <0.001	0.119
Model 3						
CC-Self-care maintenance scale	228.047 (<0.001)	35	0.591	0.474	0.159 (0.140–0.179), <0.001	0.129
CC-Self-care management scale	48.594 (<0.001)	8	0.796	0.566	0.191 (0.141–0.244) <0.001	0.072
CC-Self-care confidence scale	12.841 (<0.001)	7	0.967	0.929	0.077 (0.000–0.143), 0.214	0.049

Legend. CC: caregiver contribution; χ^2^: chi-square test; DF: Degree of Freedom; CFI: Comparative Fit Index; TLI: Tucker–Lewis Index; RMSEA: Root Mean Square Error of Approximation, SRMR: Standardized Root Mean Square Residual. Note. Model 1: Srisuk et al., 2021. Model 2: Ávila et al., 2020. Model 3: Juárez-Vela et al., 2021.

**Table 4:** Factor pattern of the Caregiver Contribution to Self-Care of Heart Failure Index (CC-SCHFI).

Caregiver Contribution to Self-Care Maintenance Scale	Factor Loading	
	Factor 1	Factor 2
1. Weigh yourself	**0.550**	0.288
2. Check your ankles for swelling	**0.480**	0.159
3. Try to avoid getting sick	**0.468**	0.086
4. Do some physical activity	0.324	**0.511**
5. Keep doctor or nurse appointments	**0.621**	0.117
6. Eat a low-salt diet	**0.686**	0.356
7. Exercise for 30 min	0.183	**0.921**
8. Forget to take one of your medicines	**0.421**	0.288
9. Ask for low-salt items when eating out or visiting others	**0.465**	0.334
10. Use a system (e.g., pill box) to help you remember your medicines	**0.707**	−0.017
11. If you had trouble breathing or ankle swelling in the past month, how quickly did you recognize it as symptoms of HF?	−0.191	**0.414**
12. Reduce the salt in your diet	0.043	**0.822**
13. Reduce your fluid intake	**0.875**	−0.187
14. Take an extra water pill	**0.754**	−0.269
15. Call the physician or nurse	−0.327	**0.559**
16. How sure were you that the remedy helped or did not help?	**0.635**	−0.141

The bold is used to highlight the values.

**Table 5:** Construct Validity.

	1	2	3	4	5	6
1. Self-care maintenance						
2. Self-care management	0.427 **					
3. Self-care confidence	0.431 **	0.479 **				
4. CC to self-care maintenance	0.341 **	0.179	0.169			
5. CC to self-care management	0.337 **	0.289 *	0.193	0.647 **		
6. CC to self-care confidence	0.333 **	0.316 *	0.314 *	0.591 **	0.646 **	
7. Caregiver preparedness	0.167	0.108	0.009	0.436 **	0.304 *	0.394 **

Legend. CC: Caregiver Contribution. Note. ** *p* < 0.001, * *p* < 0.05.

## Data Availability

The anonymized data presented in this study are available at the request of the last author. The data are not publicly available due to current personal data protection legislation.
